# Validation of the neuroconnective endophenotype questionnaire (NEQ): a new clinical tool for medicine and psychiatry resulting from the contribution of Ehlers–Danlos syndrome

**DOI:** 10.3389/fmed.2023.1039223

**Published:** 2023-05-10

**Authors:** Antonio Bulbena, Silvia Rosado, Marina Cabaleiro, María Martinez, Carolina Baeza-Velasco, Luis-Miguel Martin, Santiago Batlle, Andrea Bulbena-Cabré

**Affiliations:** ^1^Department of Psychiatry and Forensic Medicine, Universitat Autonoma Barcelona, Barcelona, Spain; ^2^Anxiety Unit, Hospital del Mar, Institute Neuropsychiatry and Addictions (INAD) CIBERSAM, Barcelona, Spain; ^3^Doctorate Program in Psychiatry, Department of Psychiatry and Forensic Medicine, Universitat Autonoma Barcelona, Barcelona, Spain; ^4^Laboratoire de Psychopathologie et Processus de Santé, Université Paris Cité, Paris, France; ^5^Department of Emergency Psychiatry and Acute Care, CHU Montpellier, Montpellier, France; ^6^Institute of Functional Genomics, University of Montpellier, CNRS, INSERM, Montpellier, France; ^7^Icahn School of Medicine, Mount Sinai, New York, NY, United States

**Keywords:** Ehlers-Danlos syndrome, anxiety, psychosomatic medicine, reliability, validity, joint hypermobility, neuroconnective endophenotype

## Abstract

**Introduction:**

The link between anxiety disorders and joint hypermobility syndrome (now under hypermobility spectrum disorders, which include hypermobile Ehlers–Danlos syndrome) has been widely replicated over the past 30 years and has grown beyond the initial nosological limits. To integrate clinical and research progress in this field, a new neuroconnective endophenotype (NE) and its corresponding instrument, the Neuroconnective Endophenotype Questionnaire (NEQ), have been developed. This new clinical construct, created with the active participation of patients, includes both somatic and psychological dimensions and symptoms and resilience items.

**Methods:**

The NE includes five dimensions: (1) sensorial sensitivity, (2) body signs and symptoms, (3) somatic conditions, (4) polar behavioral strategies, and (5) psychological and psychopathological dimensions. The NEQ information is collected through four self-administered questionnaires (sensorial sensitivity, body signs and symptoms, polar behavioral strategies, and psychological characteristics) and a structured diagnostic part that should be completed by a trained observer. This hetero-administered part incorporates (a) psychiatric diagnoses (using structured criteria, e.g., MINI), (b) somatic disorders diagnosis, using structured criteria, and (c) assessment of joint hypermobility criteria.

**Results:**

In a sample of 36 anxiety cases with 36 matched controls, the NEQ obtained high scores for test–retest, inter-rater reliability, and internal consistency. As for predictive validity, cases and controls significantly differed in all five dimensions and hypermobility measurements.

**Discussion:**

We can conclude that the NEQ has achieved acceptable reliability and validity values and, therefore, is ready to be used and tested in different samples. This original and consistent construct including somatic and mental items may improve clinical specificity, the search for more comprehensive therapies, and their genetic and neuroimaging bases.

## Introduction

After the counterintuitive finding of the strong association between joint hypermobility syndrome and anxiety disorders by our group in 1988, subsequent clinical research has confirmed this solid association between these two conditions (1).

Ehlers–Danlos syndrome was named after the observation of two dermatologists (Edvard Ehlers and Henri-Alexandre Danlos) at the beginning of the 20th century. Nowadays, it is considered a multi-systemic condition that includes a wide range of musculoskeletal features, and over the years, extra-articular symptoms, such as gastrointestinal or allergies, have gained recognition. Moreover, individuals frequently present with stress-sensitive illnesses, such as fibromyalgia or chronic fatigue syndrome (2).

There are several diagnostic methods established for identifying joint hypermobility (JHM) (3–5), the Beighton score being the most extensively used (5).

Prior to the 2017 introduction of the international criteria on the Ehlers–Danlos syndromes and hypermobility spectrum disorders, the term joint hypermobility syndrome (JHS) covered a wide group of patients some of whom had signs and symptoms that were primarily musculoskeletal (so-called hypermobility syndrome (HMS) in past terminology) and some that could equally be described as having the hypermobile type of Ehlers–Danlos syndrome (hEDS). The interchangeable use of the terms JHS and hEDS in some practices (but not all) was confusing because not all people with JHS had hEDS and many had HMS.

The 2017 international criteria introduced a much stricter definition for hEDS (6). In introducing the 2017 criteria, the term JHS was dropped. For those individuals with hypermobility-related problems that were more akin to HMS and who did not have hEDS by the 2017 criteria (or any other heritable disorders of connective tissue or other syndromic or primary cause for their condition), the term hypermobility spectrum disorder (HSD) was developed (7). Without such an inclusion, a very large, indeed the largest, and most encountered patient population with a hypermobility-related condition would not have been represented in the revised criteria.

Certainly, from a clinical perspective, many of the comorbid concerns, including anxiety disorders, that arise across the spectrum of HSD and hEDS can be considered the same both in terms of assessment and treatment (8). Currently, aiming to be pragmatic when describing past research and the relation to contemporary concepts, the term JHM is used for observations related to joint hypermobility per se, and the term HSD/hEDS (but not HSD/EDS) is encouraged for observations previously termed JHS.

The association of hEDS and anxiety disorders has also been found in other groups of psychiatric disorders, such as depression, personality disorders, eating disorders, and neurodevelopment disorders (9–12).

The overlap between HSD/hEDS, anxiety, and other psychiatric conditions can be seen from both sides: (a) How many psychiatric patients (e.g., panic and agoraphobia) meet HSD/hEDS criteria? In case–control studies, 70% of panic patients but only 15% of psychiatric controls fulfilled HSD/hEDS criteria (13). (b) How many HSD/hEDS patients meet anxiety criteria or another psychiatric diagnosis? In a retrospective study, half of the HSD/hEDS patients from a hospital sample presented psychiatric conditions, mainly mood and anxiety disorders (14). A recent review showed the growing group of disorders associated with HSD/hEDS (12). Of note, in one of the few incidence studies, a 15-year prospective follow-up, HSD/hEDS showed a relative risk of 22.3 for developing anxiety disorders, which is one of the highest risk factors described so far (15). However, it is very important not to attribute HSD/hEDS to a psychiatric origin; in fact, many HSD/hEDS symptoms are subjective and, given the lack of biological findings, these patients have often been wrongly considered “somatisers” or “functional,” which delayed the proper diagnostic process and, worse still, led to a mistaken psychiatrization (16).

The association between HSD/hEDS and anxiety disorders has also been replicated across different populations including children and elderly samples and, most recently, this association was replicated in a different species. Bowen et al. (17) found significantly higher excitability in dogs with JHM, which is a unique finding suggesting that this association may be a favorable evolutionary trait.

Anxious patients and hypermobile subjects also share other biological features, such as enhanced somatosensory perception (18, 19), and an association with functional gastrointestinal disorders (20, 21).

Despite the solid association between these clinical variables, the neurobiological substrate behind this remains unclear. Given the hereditary nature of Ehlers–Danlos syndrome, a pedigree study looking for genetic links with anxiety and phobias found a common duplication in chromosome 15; however, like multiple single-gene association studies, this finding could not be replicated (22, 23). Interestingly, both share several psychophysiological features including dysautonomia, reduced proprioception, and enhanced interoception (24). Neuroimaging studies showed some commonalities in the amygdala, insular structures, and hippocampus (25–27). In addition, different review studies also confirmed the transdiagnostic relevance of connective tissue variants with neuropsychiatric symptom expression (28, 29).

The neuroconnective endophenotype (NE) model was developed by our team to collect and assess all the somatic and psychological characteristics observed at a higher frequency in patients suffering from anxiety and other disorders and HSD/hEDS (30). The term for the new concept includes “neuro” and “connective” components, appealing both to the body-mind connectivity and connective tissue, whereas the prefix “endo” is used as the phenotype has a genetic component (HSD/hEDS).

The NE includes the following five dimensions:

1) Sensorial sensitivity, entailing, among others, high olfactory, visual and auditive sensitivity, high interoception and exteroception, low proprioception, sighing, feelings of dyspnea, and fatigue, unsteadiness, palpitations, dysphagia, urogenital or oral dynias, pain (articular or visceral) and intolerances or enhanced sensitivities to heat or cold, weather, and drugs (particularly psychotropic).
2) Body signs and symptoms include sprains and dislocations, easy bruising, especially among women, dark or blue sclerae, hypertrophic scars or keloids, dysautonomia, abdominal bloating, globus pharyngeus, ectomorphic somatotype, prolapses, and allergies.
3) Somatic conditions include irritable bowel syndrome, fibromyalgia, chronic fatigue, postural tachycardia syndrome (POTS), temporomandibular–jaw syndrome, mast cell activation, benign positional vertigo, hypothyroidism, asthma, migraine, tensional headache, cervical instability, dysfunctional esophagus, gluten intolerance, and bruxism.
4) Polar behavioral strategies. This block gathers some adaptative and defense strategies, generally identifiable by their tendency toward the two extremes of the same axis. The five axes of the model are self-others, supercontrol-uncontrol, fight-flight, avoidance-intrusion, and isolation-dependence.
5) Psychological and psychopathological dimensions (characteristics and sensations), which is a block that includes a variety of anxiety and depression symptoms and psychiatric diagnoses but also some resilience characteristics, like “*In a real emergency situation, do you quickly find solutions, that is, are you one of those who react effectively to solve it?*”; “*in moments of discussion and fighting, are you especially capable and a good warrior*?”

The relevance of the NE stems from its potential to evaluate psychiatric and psychosomatic patients in a global manner, focusing on both “mental” and “somatic” signs and symptoms. This new clinical construct has been developed with the active participation of patients and includes not only symptoms and signs but also resilience features. With the new approach afforded by NE, patients can improve their knowledge regarding their symptoms and perceptions and, subsequently, they may reach an enhanced understanding and better management of their condition. Often neglected areas, like body perception (31) and proprioception (32, 33) are particularly important in the NE, which introduces the systematic assessment of these important subjective perceptions, whose presence in both EDS subjects and anxiety patients is common and holds great potential value.

**Figure 1:**
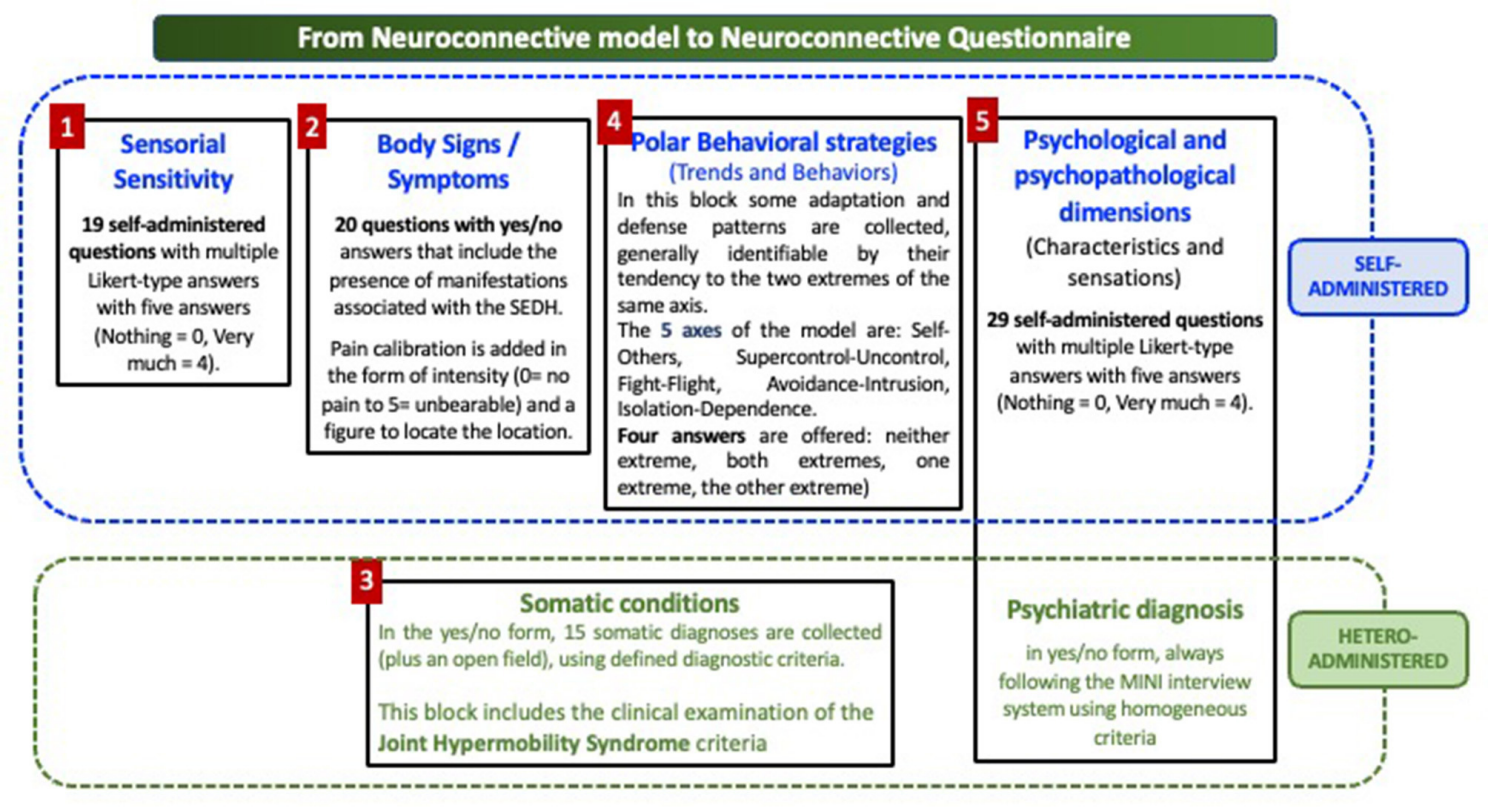
The neuroconnective questionnaire.

The Neuroconnective Endophenotype Questionnaire (NEQ) (Figure 1) was developed to assess the five dimensions of the model. NEQ information is collected through four self-administered questionnaires (sensorial sensitivity, body signs and symptoms, polar behavioral strategies, and psychological characteristics) and another structured diagnostic part that should be completed by a trained observer. This hetero-administered part incorporates (a) psychiatric diagnoses (using structured criteria, e.g., MINI), (b) somatic disorders diagnosis, using structured criteria, and (c) assessment of joint hypermobility criteria.

The sensory sensitivity scale includes 19 self-administered questions with multiple Likert-type responses with five answers (no = 0, extremely = 4).

The body signs and symptoms scale comprises 20 questions with yes/no answers that include the presence of bodily manifestations often associated with HSD/hEDS. Pain calibration is added in the form of intensity (0 = no pain to 5 = unbearable) and a figure to locate the site.

The polar behavioral strategies scale captures some adaptation and defensive patterns, generally identifiable by their tendency toward the two extremes of the same axis. The five axes of this block are as follows: me–others, supercontrol–uncontrol, fight–flight, avoidance–intrusion, and isolation–dependence. Four answers are offered: neither end, both ends, one end, and the other end.

The psychological and psychopathological dimension (characteristics and sensations) includes 29 self-administered questions with multiple Likert-type responses with five answers (no = 0, extremely = 4).

The hetero-administered component applied by a trained interviewer includes the following:

Psychiatric diagnosis in a yes/no form (categorical form), always following the MINI interview system using homogeneous criteria. Collecting lifetime prevalence is encouraged.

Somatic conditions diagnosis in a yes/no form with a total of 15 somatic diagnoses collected (plus an open field) using defined diagnostic criteria. This block also includes the clinical examination of JHM using the validated nine-item scale, which includes all Beighton criteria (34). This nine-scale substantially overlaps but the difference is that the nine-item scale requires only one side of the body to be positive, whereas the Beighton scale allows one point for each side in several items. We always use it as complementary to the Beighton scale. It is important to remember that these scales can only be assessed by clinicians who have been previously trained and validated by an expert on this condition (35).

Therefore, the main aim of this study was to calibrate the reliability and validity data of the Neuroconnective Endophenotype Questionnaire (NEQ).

## Methods

Reliability. For test–retest and inter-rater reliability measures, intraclass correlation coefficients (ICCs) were calculated for continuous variables and kappa values for categorical variables. Since different NEQ dimensions have different scoring, we calculated the sample size for each one, selecting the main objective as standard, as recommended by Bujang and Baharum (36) and Bujang et al. (37). Hence, accepting an alpha risk of 0.05 and a beta risk of 0.2 in a two-sided test, sample sizes for each estimate varied from 12 to 42 subjects to detect an estimated ICC of 0.7. To this end, between 30 and 40 participants were considered to assess reliability throughout the dimensions of the questionnaire.

Validity. Content validity (very relevant when beginning to develop an instrument) refers to the degree to which an assessment instrument is relevant to, and representative of, the targeted construct it is designed to measure. It includes face validity and construct validity. In the case of NEQ, the five neuroconnective dimensions are new concepts obtained *via* clinical observations and literature searches. Regarding face validity, this instrument took some time to develop because, after the first draft, we wanted to obtain the most specific information possible from patients and professionals. To optimize the questionnaire's content, we organized several formal and informal sessions with professionals and patients to discuss and modify each item, both at the Anxiety Unit at the Hospital del Mar and with the Asociación Nacional de Síndromes de Ehlers-Danlos e Hiperlaxitud Articular (ANSEDH). In these process meetings, both clinicians and patients contributed toward minimizing potential errors, such as underrepresentation, overrepresentation, and misrepresentation, while it is of some value to having the test appear to be valid; face validity alone is just the first step toward establishing that the test is measuring what it claims to measure. The present version (19th) includes all the items and the expressions that obtained maximum consensus. For construct validity at this early stage, Cronbach's alpha will be calculated for each scale.

ANOVAs were applied to compare case and control means, and median comparisons with Wilcoxon Z-scores were also calculated to obtain non-parametric methods.

Chi-squared tests were applied to compare rates and proportions among categorical variables between cases and control. Considering the exploratory nature of this preliminary study, correction for multiple comparisons was not applied.

For predictive validity scorings, each NEQ dimension will be compared between cases and controls. Again, the sample size was obtained *via* GRANMO (38); accepting an alpha risk of 0.05 and a beta risk of 0.2 in a two-sided test, a minimum of 26 participants per group were necessary to recognize a difference greater than or equal to 8 units as statistically significant. The common standard deviation was assumed to be 10 and a drop-out rate of 5% was anticipated.

Informed consent was obtained from each participant and the protocol was approved by the local Ethical Committee.

## Results

**Sample**, a total of 38 patients from the Anxiety Unit at the Hospital del Mar (Parc de Salut Mar, Barcelona) and 38 matched controls were invited to participate in the study; informed consent was obtained from each participant before entering the study once the technical and ethical conditions were properly accepted. Two patients dropped out, one for minor surgical procedures and another because they moved away during the study, therefore, the final sample was 36 participants.

Controls were healthy volunteers who agreed to participate in the study after being contacted by email and summoned afterward in person. Inclusion criteria included those aged between 18 and 75 years and residing in the province of Barcelona. All participants gave their informed consent before starting the study. Exclusion criteria for controls included the presence of active anxiety disorder, or in remission, assessed *via* the MINI, any form of cognitive impairment, and musculoskeletal conditions or muscle spasticity secondary to neuromuscular diseases that hindered the correct evaluation of joint laxity.

The case group consisted of 24 women and 12 men, whereas 21 women and 15 men were in the control group. Cases and controls did not differ by gender (χ^2^ = 0.534; *p*-value = 0.46) or age (cases: mean (±SD) 42.13 years (± 11.9); controls: mean (±SD) 45.69 years (±18.9)) (ANOVA F = 0.9; *p* = 0.34).

**Internal consistency**, measured by Cronbach's alpha, was 0.866 for the sensorial sensitivity 19-item scale, 0.763 for the Body signs and symptoms 20-item scale, 0.876 for the psychological characteristics-sensations 30-item scale, 0.724 for polar behavior strategies 5-item scale, and 0.792 for the hypermobility 9-item scale (Table 1).

**Table 1:** Reliability neuroconnective questionnaire NEQ.

	**Alpha Cronbach**	**Intraclass corr. coefficient**	Kappa coefficient^*^
Sensorial sensitivity (19 item)	0.861	0.986	-
Body signs and symptoms (20 item)	0.763	-	0.6–1
Somatic conditions (19 item)	-	-	0.45–1
Polar strategies (5 item)	0.724	0.8–1	-
Psychological dimensions (30 item)	0.876	0.996	
Psychiatric diagnosis (20 item)	-		0.65–1
Hypermobility score (9 item)	0.792	0.967	-

* Kappa values are obtained for item yes/no.

**Reliability** (test–retest) was calculated for each item of the five scales and the total scale. ICC was established for self-administered continuous scales in the form of test–retest with an interval of (16 ± 2) days, whereas Cohen's kappa was calculated for categorical items (i.e., yes/no). Sensorial sensitivity and psychological scales obtained ICC coefficients higher than 0.9. For body signs and symptoms, kappa values ranged between 0.6 and 1, whereas for polar behavior strategies, the range was between 0.8 and 1.

**Inter-examiner reliability** was also calculated for hypermobility items obtaining a range of ICC values between 0.7 and 1.

**Psychiatric diagnoses**, evaluated *via* MINI, also obtained a high degree of concordance between examiners, with kappa values between 0.65 and 1. Medical diagnosis applying the criteria list *ad hoc* to each diagnostic label obtained kappa values of 0.45 to 1. The lowest value was found for the cyclic vomit item, which prompted authors to review this criterion.

### Cases and controls

Anxiety cases and controls were compared in every dimension of the neuroconnective model. Most sensorial items scored significantly higher in the anxiety cases but none scored higher in the control group; those items not reaching significance were the least prevalent in both samples. The total sensory scale showed significantly higher scoring for the anxiety cases [mean (±SD) = 33.19 (±11.5)] than the controls, [mean (±SD) = 14.77 (±5.98)] (ANOVA F:72.58 *p* < 0.0001).

In the body signs and symptoms scale, 12 of the 20 items were significantly higher among the anxiety cases, but none scored higher in the control group. Again, those not reaching significance were the least prevalent in both samples. The total body signs and symptoms scale scored significantly higher for the anxiety cases (mean (±SD) 7.86 (±2.88)) than for the controls [(mean (±SD) 4.22 (±3.39)] (ANOVA F 12.033 *p* = 0.0011).

In the psychological characteristics scale, 23 of 31 items were significantly higher among the anxiety cases. As expected, most items reflecting psychopathological traits showed significant differences between groups. On the other hand, this scale also contains items reflecting some advantageous or positive traits and most of them scored higher among the anxiety cases; however, they did not reach statistical significance. The entire psychological scale scored significantly higher in the anxiety cases, with a mean (±SD) of 48.61 (±17.90) than in controls, with a mean (±SD) of 27.61 (±10.55) (ANOVA 36.757, *p* < 0.0001).

In polar behavioral axes, the anxiety cases showed significantly higher polar tendencies than controls on four of the five axes: supercontrol/lack of control (χ^2^ = 11.364; *p* = 0.0099), dependency/isolation (χ^2^ = 25.768; *p* = <.0001), fight /flight (χ^2^ = 17.068; *p* = 0.0007), and me/others (χ^2^ = 9.085; *p* = 0.002), whereas avoidance/ intrusion (χ^2^ = 6.299; *p* = 0.0979) did not reach statistical significance. Total scoring for the polar behavior scale was significantly higher for the anxiety cases [mean (±SD) 6.63 (±2.77)] than for controls [mean (±SD) 3.50 (±2.71)] (ANOVA F 23.63 *p* < 0.0001) (Table 2).

**Table 2:** NEQ comparison cases and controls.

	**CASE**	**Median**	**CONTROL**	**Median**	**F**	***p***	**Z**	***p***
**Sensory sensitivity**	33.19 (±11.5)	32	14.77 (±5.98)	14	72.58	0.0001	6,351	<0.0001
**Body signs and symptoms**	7.86 (±2.88)	7	4.22 (±3.39)	3.5	12.33	0.0011	3,474	0.0005
**Psychological characteristics**	48.61 (±17.90)	46	27.61 (±10.55)	24	36.76	<0.0001	51,001	<0.0001
**Polar behavior strategies**	**CASE (** ***n*** **)**	%	**CONTROL (n)**	%	**x** ^2^	*p*		
Both supercontrol and lack of control	14	38.9	4	11.11	11,364	0.0099		
None supercontrol nor lack of control	8	22.22	20	56.56				
Only one of them	14	38.9	12	33.33				
Both dependent and isolated	29	80.56	11	30.56	25,768	<0.0001		
None dependent nor isolated	6	16.67	18	50				
Only one of them	1	2.78	7	19.4				
Both avoidance and intrusion	12	33.33	5	13.89	6,299	0.0979		
None avoidance nor intrusion	20	55.56	25	69.44				
Only one of them	4	11.11	6	16.67				
Both fight and fly	24	66.67	10	27.78	17,068	0.0007		
None fight nor fly	6	16.67	20	55.56				
Only one of them	6	16.67	6	16.67				
Both me and others	25	69.44	13	36.11	9,085	0.0282		
None me nor others	5	13.89	14	38.89				
Only one of them	6	16.67	9	25.01				

Eight of the 13 medical diagnoses (irritable bowel syndrome, functional dyspepsia, somatoform vertigo, chronic fatigue syndrome, fibromyalgia, hypothyroidism, asthma, and temporomandibular-mandibular syndrome) were significantly more prevalent in the anxiety case group. However, dysfunctional esophagus, postural orthostatic tachycardia syndrome, tensional headache, migraines, multiple chemical sensitivity (not just intolerances or allergic reactions), gluten intolerance, and intolerance to some medications did not reach statistical significance.

Psychiatric diagnoses were only found in the anxiety case group, as expected, although one control appeared to have an unclear history of a bipolar episode in their youth.

The anxiety cases [mean (±SD) 4.50 (±2.72)] scored significantly higher in the hypermobility scale than the controls [mean (±SD) 2.50 (±2.15)] (ANOVA F = 11.94, *p* = 0.0009), as they did for most of the individual items of the scale. In the absence of a gold standard for Ehlers–Danlos, it is mandatory to calculate different cut-off points to obtain different degrees of prevalence. Hence, after applying different cut-off points to this scale, results show significant differences in every grouping. In the 3/4 cut-off, there were 24 cases (66.67%) and 8 controls (22.22%) classified as hypermobile, χ^2^ = 14.955, *p* = 0.0001. In the 4/5 cut-off, there were 19 cases (52.78%) and 7 controls (19.44%) classified as hypermobile, χ^2^ = 8.921, *p* = 0.0028. In the 5/6 cut-off, there were 15 cases (41.67%) and 4 controls (11.11%) classified as hypermobile, χ^2^ = 9.082, *p* = 0.0026. In every cut-off, the obtained “case” groups meet modern criteria for HSD/hEDS.

## Discussion

The instrument proposed for this new clinical endophenotype has shown good psychometric standards of reliability and validity. Internal consistency has achieved acceptable figures for each scale. Test–retest reliability was quite satisfactory since most coefficients were above 0.8, and the inter-examiner kappa values obtained were within a similar range.

In the comparison between the anxiety cases and controls, each NEQ scale obtained significantly higher scoring. The sensory scale score was higher among the anxiety cases, which confirms previous findings by different groups (39–41), and in a non-clinical population using the 10-item Barsky's Somatosensory Amplification Scale (SSAS) (42). We decided to develop a new list of items following proposals from patients that allowed us to capture more specific perceptions.

The body signs and symptoms scale was significantly higher among the anxiety cases, reflecting the value of considering these somatic characteristics and clues among anxiety patients. Most of these are closely related to Ehlers–Danlos features and it is very important not only to assess them in patients with anxiety but to especially avoid premature misattribution to psychogenic causation or even to the blurry somatization group now renamed as somatic symptom disorder in DSM 5. Psychological factors and precipitants may be present, however, acknowledging these often-neglected HSD/hEDS symptoms will provide new clues to better understand patients.

The psychological (characteristics and sensations) scale includes a variety of the old “neurotic ambit” items, such as anticipatory anxiety, worry, social anxiety, fears, phobias, health anxiety, and functional neurologic or gastrointestinal symptom, together with other resilience features also suggested by the patient groups. This is a new line for clinical assessment, often only centered on negative items, whereas items related to clinical anxiety scored significantly higher in patients, some related to resilience, albeit always higher in patients, did not reach significance. This may be due to sampling reasons as the cases seen in a country-reference anxiety unit might include more severe cases, which may hamper obtaining more positive answers.

The polarized behavior scale is based on several basic adaptive strategies that can be represented between two poles: overcontrol/lack of control, dependency/isolation, fight/flight, me/others, and avoidance/ intrusion. These are tendencies that have been related to personality patterns as well as clinical conditions, particularly anxiety. The hypothesis was that patients would show more polarized tendencies either uni-directionally (43–46) or bi-directionally, along the lines of coexisting dichotomies like altruism/ egoism (47) and overcontrol/uncontrol (48, 49).

The results obtained confirmed that patients with anxiety showed a greater tendency than the controls toward these polarized behaviors. These preliminary findings provide grounds to include this scale in the phenotype. One of the potential advantages of this axis is that it provides psychological clues that can guide psychotherapeutic approaches, as has been the case for overcontrol/uncontrol with radically open dialectical behavior therapy (50).

One important contribution of the neuroconnective endophenotype is the systematic assessment of somatic conditions, which were found to be prevalent, either among anxiety disorders or in HSD/hEDS. The patients proposed quite a few clinical conditions; however, the final list included 15, which were properly defined by written diagnostic criteria.

In the comparison between the anxiety cases and the controls, only half of these somatic ailments (irritable bowel, functional dyspepsia, somatoform vertigo, chronic fatigue, fibromyalgia, asthma, and temporomandibular–jaw syndrome) were significantly more common among the anxiety cases; however, general frequencies were low. Most of them had been related to anxiety but not the joint hypermobility spectrum.

As expected, the prevalence of hypermobility was again found to be significantly higher among the patients with anxiety, which confirms previous research. Even applying different cut-off points, these differences remained significant. Albeit prevalence was not an objective of this study, the figures obtained (66% in the 3/4 cut-off point and 53% in the 5/6 cut-off point) agreed with previous publications (13, 19).

This study has several limitations. First, from the early joint hypermobility syndrome in the 80s, this syndrome has been redefined, particularly after the historical EDS meeting in New York in 2016 where new nosological approaches were introduced, such as the hypermobility spectrum disorder (HSD). This produces some difficulties to reconcile past and present papers, although there is intense overlap among most criteria. In this case, we have taken the advice to collect them under the heading HSD/hEDS which provides strict specificity. Second, sample sizes, although appropriate for reliability studies, were relatively small (but properly calculated) to compare the anxiety cases and the controls. More cases and controls would be necessary to figure out the value of each item and each scale and to carry out the necessary multivariate analysis to ascertain the predictive validities of NEQ. Third, an assessment of somatic conditions is made via an interview with the participant, which may be affected by recall bias. To palliate this bias, medical records of cases and controls were screened with the permission of each study participant. Fourth, anxiety cases included only patients diagnosed in our panic, agoraphobia, social phobia, specific phobia, and generalized anxiety disorder units. In this sample, there were no cases of obsessive-compulsive disorder or stress-related disorders. Fifth, cases were taken from an anxiety unit, which may represent a more severe sample. However, in different studies to date, most results concur. Sixth, no specific anxiety or depression scales were applied to the cases and controls. This procedure will be performed in the next validation study, where each scale will be analyzed in terms of concurrent validity with selected scales. Seventh, to apply NEQ, some training will be necessary and, therefore, one should not use it without proper (although brief) training to be able to assess joint hypermobility.

Four points prompted us to develop this Neuroconnective Endophenotype Questionnaire. First, the extensive research on the association between HSD/hEDS and anxiety has been overwhelmingly replicated. These patients suffer the clinical manifestations of both disorders, but they often receive only one of the two assessments and treatments. Furthermore, it is well known that HSD/hEDS is a systemic condition well beyond the musculoskeletal system, also affecting gastrointestinal, neurological, immuno-allergology, gynecological, and urological systems, among others. Since the association with HSD/hEDS is so extensive, it is likely that those patients suffering from anxiety may also suffer from the clinical conditions of these systems. Second, present nosological approaches do not allow the concurrent inclusion of both mental and somatic conditions together. Instead, the approaches both of DSM and ICD take the direction of causality, and emphasis is placed on the secondary appearance, but not on coexistence. Examples of these views are DSM 5: 316 *Psychological Factors Affecting Other Medical Conditions* 316 (F54) or, in the inverse direction, Anxiety Disorder Due to Another Medical Condition DSM-5 293.84. Some isolated but interesting proposals like ALPIM (anxiety, laxity, pain, immune, and mood) have been described along the lines of comorbidity (51).

Third, since most patients show mixed subjective and objective manifestations, in most instances their own words could better capture this clinical information. The NEQ has tackled the challenge of the patient's participation and this questionnaire was constructed with the active cooperation of patients, who suggested and corrected most items, which is a significant step toward clinical validity.

Fourth, a new endophenotype requires proper psychometric assessment, and the first step is to develop an instrument with good reliability and proper validity measures. The development period took some time because the authors wanted to maximize face validity, given the original and transversal nature of the proposal. After many sessions with patients, professionals, and patient associations, the present version was considered ready for calibration. This study deals with the Spanish version of the NEQ, but English, French, and Catalan versions are on the way. Also, cultural validation will be made with the Spanish and Colombian Spanish versions.

After almost 30 years of successful research on the association between joint hypermobility and anxiety disorders and other psychiatric conditions, the subsequent step was to identify a core endophenotype that can gather these clinical features. This endophenotype might afford more strength to clinical practice and research. For patients, the acknowledgment of this broad spectrum makes them able to realize the mixed nature of their condition; in our experience, this results in great relief and understanding for patients. For psychiatrists and mental health workers, assessment and treatment of both somatic and mental symptoms can be a relevant step toward a better understanding of these patients' suffering. Considering that both anxiety disorders and Ehlers–Danlos hypermobility types have strong heritability without any specific genetic testing as yet, engaging in new research with this more comprehensive endophenotype might provide new grounds to obtain improved specificity of the genetic basis. The originality of NEQ is that it includes both objective and subjective features, both patient and professional contributions, both somatic and psychological features, and both negative and positive (resilience) features.

This study is the necessary first step toward the full validation process of a new clinical construct. We envisage larger and varied samples to determine cut-off points and extensive predictive validity studies will lead to biological studies (i.e., genetic and neuroimaging) to obtain external validity. Also, NEQ will need to be validated in bigger HSD/hEDS samples and common comorbid conditions, such as fibromyalgia, chronic fatigue syndrome, mast cell activation disorders, and postural orthostatic tachycardia syndrome, as well in those psychiatric conditions that have shown high HSD/hEDS prevalence.

Most importantly, therapeutic trials, beyond psychopharmacological approaches, need to be carried out. Perhaps the most promising path is the design of preventive programs for children with this phenotype, which we anticipate should contain educational, psychological, and physical components. NEQ has obtained acceptable figures regarding reliability, internal consistency, and both face and predictive validity in the comparisons with matched controls; it is ready to be used and tested. To carry out this study in children, we envisage developing a specific children's version of the NEQ, which, as yet, is in the early stages.

## Data availability statement

The raw data supporting the conclusions of this article will be made available by the authors, without undue reservation.

## Ethics statement

The studies involving human participants were reviewed and approved by Comité de Ética de la Investigación con medicamentos del Parc de Salut MAR. The patients/participants provided their written informed consent to participate in this study.

## Author contributions

AB, SR, and AB-C contributed to the conception and design of the study. SR, CB-V, MM, MC, L-MM, and SB carried out assessments, fieldwork, and contributed to the first draft and first version of the manuscript. AB and AB-C performed the revision of the manuscript. All authors contributed to the manuscript revision, and have read and approved the submitted version.
