# Corrigendum: Validation of the neuroconnective endophenotype questionnaire (NEQ): a new clinical tool for medicine and psychiatry resulting from the contribution of Ehlers–Danlos syndrome

**DOI:** 10.3389/fmed.2023.1338616

**Published:** 2023-12-07

**Authors:** Antonio Bulbena, Silvia Rosado, Marina Cabaleiro, María Martinez, Carolina Baeza-Velasco, Luis-Miguel Martin, Santiago Batlle, Andrea Bulbena-Cabré

**Affiliations:** ^1^Department of Psychiatry and Forensic Medicine, Universitat Autonoma Barcelona, Barcelona, Spain; ^2^Anxiety Unit, Hospital del Mar, Institute Neuropsychiatry and Addictions (INAD) CIBERSAM, Barcelona, Spain; ^3^Doctorate Program in Psychiatry, Department of Psychiatry and Forensic Medicine, Universitat Autonoma Barcelona, Barcelona, Spain; ^4^Laboratoire de Psychopathologie et Processus de Santé, Université Paris Cité, Paris, France; ^5^Department of Emergency Psychiatry and Acute Care, CHU Montpellier, Montpellier, France; ^6^Institute of Functional Genomics, University of Montpellier, CNRS, INSERM, Montpellier, France; ^7^Icahn School of Medicine, Mount Sinai, New York, NY, United States

**Keywords:** Ehlers-Danlos syndrome, anxiety, psychosomatic medicine, reliability, validity, joint hypermobility, neuroconnective endophenotype

In the published article, there was an error regarding the affiliations for Silvia Rosado. As well as having affiliation 2, they should also have “Doctorate Program in Psychiatry, Department of Psychiatry and Forensic Medicine, Universitat Autonoma Barcelona, Barcelona, Spain”. Silvia Rosado's affiliations are now 2 and 3.

The authors apologize for this error and state that this does not change the scientific conclusions of the article in any way. The original article has been updated.

