# Supplementary material for: Validation of the neuroconnective endophenotype questionnaire (NEQ): a new clinical tool for medicine and psychiatry resulting from the contribution of Ehlers–Danlos syndrome
Source: Front Med (Lausanne). 2023 May 10;10:1039223. doi: 10.3389/fmed.2023.1039223 (PMC10206165; doi:10.3389/fmed.2023.1039223)
Supplement: Supplementary file 1 [file Data_Sheet_1.pdf]

NEUROCONNECTIVE ENDOPHENOTYPE QUESTIONNAIRE v20

Name……………………………………………………………………………………….Age………

Female ☐ Male ☐ Date……. /………. /202……

Often people with medical problems (which may also include anxiety or depression) have characteristics, manifestations, or physical symptoms that are not properly identified. **In this questionnaire, we will ask you about these characteristics to understand everything that affects you and your experiences at the body level and some symptoms of the mind.**

The neuroconnective phenotype was developed to understand the physical symptoms or psychological traits of people who suffer from these issues. This phenotype has 5 parts: sensory sensitivity, physical signs, somatic conditions, as well as some psychological-psychiatric aspects and associated behaviours.

**Sensory sensitivity**	No	A little	Quite	Very	Extremely
1-Do you think that you have a sensitive nose and are very capable of noticing odours (especially bad ones)?	0	1	2	3	4
2- Do you think that you are sensitive to noise? (Does it bother you?)	0	1	2	3	4
3 - Do you consider yourself a very sensitive person in general?	0	1	2	3	4
4 - Do you find it difficult or uncomfortable to look into the eyes of others or maintain your gaze when they look at you?	0	1	2	3	4
5 - In periods when the light decreases (e.g., winter) do you notice changes in your energy or mood?	0	1	2	3	4
6 - Do you find that changes in the weather (e.g., cloudy days, before it rains, etc.,) affect your mood?	0	1	2	3	4
7 - Do you ever feel unsteadiness, dizziness, or insecurity when walking? (e.g., like walking on a boat or as if walking on clouds)	0	1	2	3	4

8 - Do you think that heat or cold particularly affect you? (Circle which one(s))	0	1	2	3	4
9 - Do you feel heavy or bloated after meals?	0	1	2	3	4
10- Do you find yourself sighing often?	0	1	2	3	4
11 - Do you easily sense your body, your breathing, your bowel movements, or your pulse?	0	1	2	3	4
12 - Do you notice that your heart is sometimes racing/beating too fast?	0	1	2	3	4
13 - Do you feel like you are choking or short of breath in times of stress?	0	1	2	3	4
14 - Do you feel a lump or pressure in your throat in times of stress?	0	1	2	3	4
15- Do you have pain in the cervical vertebrae?	0	1	2	3	4
16 - Do you have pain in the lumbar vertebrae?	0	1	2	3	4
17 - Have you ever felt pain/burning in your mouth or pain/burning in the genital area?	0	1	2	3	4
18 - Do you feel that medication in general (even small doses) has a strong effect or that you don't tolerate it?	0	1	2	3	4
19 - Have you ever had discomfort related to the use of soaps, jewellery, or other chemicals?	0	1	2	3	4
TOTAL					

**Body signs/symptoms**	YES	NO
1 - Have you noticed that you bruise at the slightest injury or that you have bruises when you do not remember hurting yourself?	1	0
2 - Do you have a slightly raised or slightly enlarged scar?	1	0
3 - Have your hands or feet ever become swollen while walking for a while, spending time in stores, or standing in line for a while?	1	0

4 - Have you ever had a fainted (syncope) (with weakness, sweating, etc.,)?	1	0
5- Do you notice that you sometimes have a hard time swallowing or that you almost choke on food?	1	0
6 - Is your way of walking sometimes unsteady, clumsy, and even stumbling?	1	0
7 - Have you suffered sprains or dislocations? Indicate	1	0
8 - Have you had temporomandibular pain (in front of the ears) or bruxism (clenching teeth when sleeping)? Circle which please.	1	0
9- Is your sleep often not restful and you wake up tired?	1	0
10 -Have you had hernias (inguinal, umbilical, hiatus ...)? Indicate	1	0
11 - At the end of the day, do you have back pain when you lie down to rest?	1	0
12 - Do you have acid reflux after meals or when getting out of bed?	1	0
13 - Do you tend to have periods of increased bowel movements?	1	0
14 - Do you tend to have periods of increased urination?	1	0
15 - Do you have episodes of fear of losing urinary control?	1	0
16 - Have you suffered from retinal detachment or lens dislocation?	1	0
17 - Do you have premenstrual syndrome? (e.g., pain, bad mood, sadness, anxiety, etc.)	1	0
18 - Have you been diagnosed with a mitral valve murmur or prolapse (e.g., uterus, rectum, etc.,)?	1	0
19 - Have you had carpal tunnel syndrome or pneumothorax? (circle which one(s))	1	0
20 - Do you have a family member who suffers from some of these symptoms or similar?<br>Please describe	1	0
TOTAL		
Observations		

1 - Locate and assess your pain in the last 30 days

2 - Only in case you need another figure - Locate and assess your pain in the last

PAIN SEVERITY

Painless	0
Mild	1
Discomfort	2
Stressful	3
Horrible	4
Unbearable	5

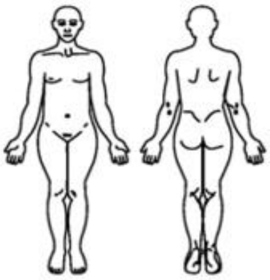

E=External
I=Internal

PAIN SEVERITY

Painless	0
Mild	1
Discomfort	2
Stressful	3
Horrible	4
Unbearable	5

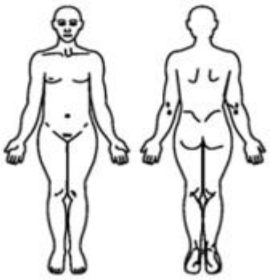

E=External
I=Internal

Characteristics and Sensations	No	A little	Quite	Very	Extremely
1 - Do you often have difficulty sleeping? falling asleep? Wake up during the night? (Circle which one(s))	0	1	2	3	4
2 - Do you have difficulty entering enclosed places, e.g., elevators, subways, planes?	0	1	2	3	4
3 - Do you have difficulty going to crowded places?	0	1	2	3	4
4 - Do you find it difficult to wait, stand in line, or take turns?	0	1	2	3	4
5 - Have you ever felt strange, as if you were not yourself?	0	1	2	3	4
6- Are you usually a very active person, always doing things and find it difficult to not be doing something?	0	1	2	3	4
7 - Are you strongly affected by losses, for example, death of a parent, friends, pet, loss of a partner, etc?	0	1	2	3	4
8 - Are you very sensitive to being rejected, being ignored, not feeling taken into account or abandoned?	0	1	2	3	4
9 - Would you define yourself as a worrier?	0	1	2	3	4
10 - Do you tend to anticipate the worst and think about bad things?	0	1	2	3	4
11 - In a real emergency situation, do you quickly find solutions, that is, are you one who reacts effectively to solve it?	0	1	2	3	4

12 - At times of discussion and fighting, are you especially capable and a good warrior?	0	1	2	3	4
13 - Do you tend to "see" or "detect" things before others?	0	1	2	3	4
14 - Does your environment (e.g., friends) consider you to be successful in your studies, work, or family?	0	1	2	3	4
15 - Do you think you have been especially able to overcome life's obstacles?	0	1	2	3	4
16 - Are you usually quicker and more decisive than others?	0	1	2	3	4
17 - Do you tend to spend a lot of time in the shower or toilet?	0	1	2	3	4
18 - Do you tend to wash your hands a lot?	0	1	2	3	4
19 - Do you feel intense discomfort or rejection of your body secretions (e.g., urine, faeces, saliva)?	0	1	2	3	4
20 - Have you had thoughts of hurting someone or killing or fear of such thoughts?	0	1	2	3	4
21 - Do you have tendencies to check if you have closed the door, turned off the gas, appliances, etc.?	0	1	2	3	4
22 - Do you tend to fear illnesses or are very fearful for your health when you notice any symptoms?	0	1	2	3	4
23 - Do you have a tendency to count numbers (like counting license plates) or to make lists?	0	1	2	3	4
24 - Are you apprehensive about taking medication when a doctor prescribes it?	0	1	2	3	4
25 - Do you consider yourself a compulsive shopper?	0	1	2	3	4
26- Have you had moments when you have become speechless?	0	1	2	3	4
27 - Have you had episodes of paralysis in the arms or legs?	0	1	2	3	4
28 - Have you had rare episodes of seizures (e.g., non-epileptic) or strange tremors?	0	1	2	3	4

29 - Have you ever spent minutes or hours in a state of mental disconnection?	0	1	2	3	4
30 - Have you ever had episodes for minutes or hours with no memory?	0	1	2	3	4
31 - Have you had periods of sadness and depression lasting more than 10 days?					
TOTAL					

Trends and Behaviours

To understand the questions, it may be helpful to look at the diagram below, examining each axis and its extremes.

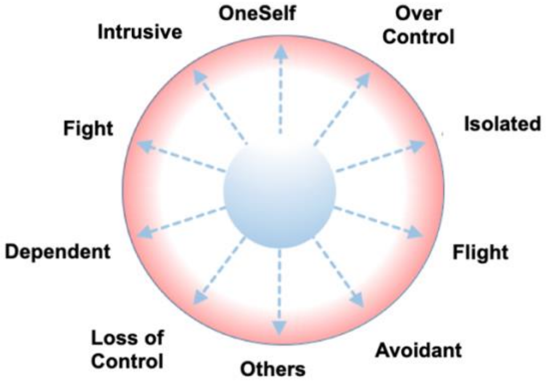

There are people who may present apparently contradictory behaviours that appear as if they were automatic dimensions of their character. Often, there are very personal, intimate profiles specific to each person and sometimes others only see one of the dimensions.

In fact, we want to know whether you exhibit these two polarities. Therefore,

1 - Supercontrol/Lack of control. Do you tend to supercontrol your things and situations (e.g., order, cleanliness, comply with rules, etc.), while, other times you find yourself doing the opposite (disorder, lack of control, etc.)? *Check ONE of the four answers.*

I have neither supercontrol nor lack of control	
I certainly tend to behave on these two poles / My control oscillates between too much or too little	
I tend toward lack of control	
I tend to supercontrol	

2 - Dependency/Isolation. Do you sometimes find yourself to be quite dependent on someone or a group, while other times you isolate yourself, withdraw, or hide from others? *Check ONE of the four answers.*

I do not consider myself a dependent person or a person who tends to withdraw.	
I certainly oscillate between being dependent at times and isolating myself at others times.	
I only tend to be dependent	
I only tend to isolate myself	

3 - Avoidance/Intrusion. Do you sometimes find it difficult to speak or express yourself with others, but when you do express yourself, you do so with a certain vehemence or even anger? *Check ONE of the four answers*.

I do not consider myself a person who finds it difficult to express themself or speak with others, or who expresses themselves vehemently.	
I tend to behave on those two poles: either I do not speak or I do so with annoyance.	
I only tend toward Avoidance	
I only tend toward Intrusion	

4 - Fight/Flight. Are you sometimes a fighter and incisive, while at other times, you prefer to flee, escape, or simply avoid conflict? *Check ONE of the four answers*.

I do not consider myself a fighter or one who avoids conflict	
I certainly tend to oscillate between a fighter and active while at other times I become evasive.	
I only tend towards fighting/aggression	
I only tend towards evasion or escape	

5 - Myself/Others. Do you sometimes find that your dedication to others is very great (family, work, etc.,) while, at other times you withdraw into yourself and want to be left alone? *Check ONE of the four answers.*

I do not consider myself a person who dedicates either fully to others or to myself.	
I certainly oscillate between dedicating myself to others or withdrawing depending on the moment.	
I only tend to be dedicated to myself	
I only tend to dedicate myself to others	

DIAGNOSIS - Somatic symptoms

**DIAGNOSTICS** **(fill in with the doctor or nurse)**	**YES**	NO
1 - Irritable bowel syndrome?	1	0
2 - Dysfunctional oesophagus?	1	0
3 - Vertigo?	1	0
4 - Postural orthostatic tachycardia syndrome?	1	0
5 - Chronic fatigue?	1	0
6 - Fibromyalgia?	1	0
7 - Migraines?	1	0
8 - Hypothyroidism?	1	0
9 - Asthma?	1	0
10 - Multiple Chemical Sensitivity? *(not just intolerances or allergic reactions)*	1	0
11 - Gluten intolerance?	1	0

12 - Intolerance to some medications? Detail:	1	0
13 - Temporomandibular joint syndrome?	1	0
14 - Observe asthenic (frail, long-limbed, narrow-chested physique)/ectomorphic biotype?	1	0
15 - Dark sclera (somewhat dark)	1	0
OTHER DIAGNOSES:	1	0
TOTAL		

DIAGNOSIS: Psychiatric disorders

**PSYCHIATRIC DIAGNOSTICS (fill in with the doctor or nurse)**	**YES**	NO
1 - Major depressive disorder? (F32)	1	0
2 - Bipolar disorder (531)	1	0
3 - Anorexia Nervosa? (F50.0)	1	0
4 - Bulimia Nervosa? (F50.2)	1	0
5 - Binge Eating Disorder? (F50.8)	1	0
6 - Obsessive-compulsive disorder? (F42.)	1	0
7 - Functional Neurological Symptoms Disorder (T. Conversion) (F44)	1	0
8 - Generalized anxiety disorder? (F41.1)	1	0
9 - Panic Disorder? (F41.0)	1	0
10 - Hypochondria? Somatic Symptom Disorder? (F45.1)	1	0
11 - Social Anxiety? (F40.1)	1	0
12 - Agoraphobia? (F40.0)	1	0
13 - Specific phobia? (F40.2)	1	0
14 - Borderline Personality Disorder? (F60.3)	1	0
15 - Schizophrenia? (F 20)	1	0
16 - Hyperactivity Disorder (ADHD)? (F90)	1	0
17 - Alcohol Use Disorder? (AUD) (F10)	1	0
18 - Substance Use Disorder? Marijuana (F12) - Cocaine- (F14)	1	0
OTHER PSYCHIATRIC DIAGNOSES (Autism Spectrum disorder, etc)	1	0
TOTAL		

ANNEX: CRITERIA FOR THE CLINICIAN

HYPERMOBILITY ASSESSMENT GUIDELINE

Name..Date / / 2....

Age............ Male □ Female □ Evaluator ....

Item			Right	Left
1	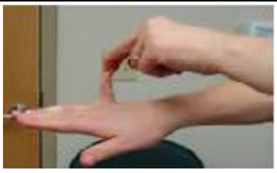	**90° flexion of the little finger**. With the palm of the hand on a base, it should be bent upwards more than 90°.<br><br>If the third phalanx can bend by more than 90º, it fulfils the criterion.	★	★
2	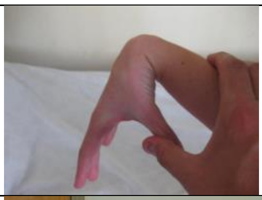	**Thumb flexion**. With the flexed downwards, you can almost touch the forearm with your thumb.<br><br>It is also positive if the thumb is horizontal rather than parallel to the forearm.	★	★
3	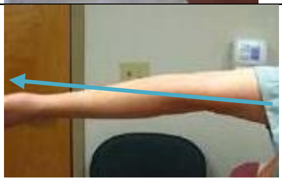	**Hyperextension of the elbow**<br>With the arm extended and the elbow downwards. The elbow extends 10° or more.<br>Some arms are not straight, first place the arm so that it is straight (flat), and then check the item.	★	★
4	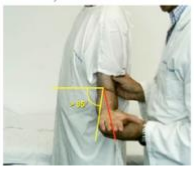	**Shoulder rotation**<br>With the arm against the body and holding the biceps with the opposite hand, the arm should rotate back 85°.<br>Hold your forearm firmly without touching your elbow and help rotate your arm gently from your wrist.		
5	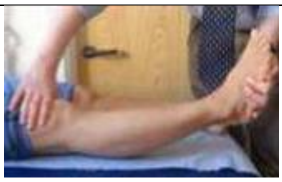	**Hyperextension of the knee of more than 10°**<br>Lying on a flat surface with the knee straight. The evaluator places one hand on the knee and with the other tries to raise the leg. It should rise 10° or more.<br>Sometimes you can already see it with the naked eye while the person is standing.	★	★
6	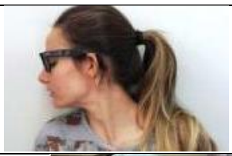	**Head rotation**<br>Rotate the head to one side at an angle close to 90°.<br>The chin is aligned/above the shoulder. You can touch the shoulder with your chin (Attention, do not raise the shoulder!)		
7	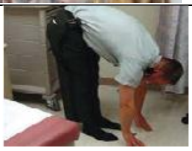	**Spinal flexion**<br>Standing, trunk flexed with the knees fully extended until touching the ground with the palms of the hands. The legs may be a little apart and it is not necessary for the entire palm to touch the ground, the phalanges can touch.	★	
8	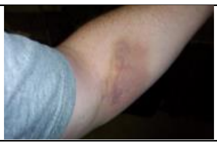	Easily presents **ecchymosis** (bruises).		
9	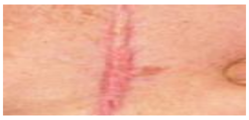	**Hypertrophic and/or keloid scars.**		
★ **BEIGHTON Criteria** ☐		**Hypermobility criteria HLX9 TOTAL**<br>(only one point (not two) when either side is positive)	☐	

OPTIONAL

Self reported Hypermobility questionnaire (Bulbena, Antonio, Mallorquí-Bagué, Núria, Pailhez, Guillem, Rosado, Silvia, González, Ignacio, Blanch-Rubió, Josep, & Carbonell, Jordi. (2014). Self-reported screening questionnaire for the assessment of Joint Hypermobility Syndrome (SQ-CH), a collagen condition, in Spanish population. *The European Journal of Psychiatry*, *28*(1), 17-26. https://dx.doi.org/10.4321/S0213-61632014000100002)

Collagen Elasticity Questionnaire (S-SHC)

INSTRUCTIONS: Collagen is a protein that forms part of the skin, bones, tendons, and connective tissue. This questionnaire contains 7 simple questions to discover the degree of elasticity of the collagen fibres in your body.
Please fill out the form and read the questions carefully, then circle the answer (Yes/No) that you consider most accurate according to your experience.

As a child (>6) could you, or have you ever been able (even today) to place your palms on the floor without bending your knees?

YES - NO

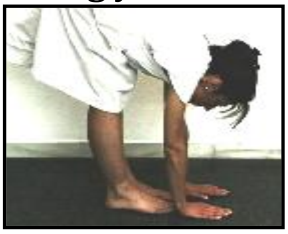

1 - Can you bend your thumb to the forearm in the following way? (e.g., left hand)

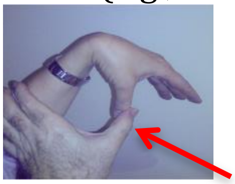

YES - NO

2 - As a child (>6), could you open your legs like in the photo or contort your body into some special postures? (See some examples; you might have one of your own) **Any one of them counts as positive.**

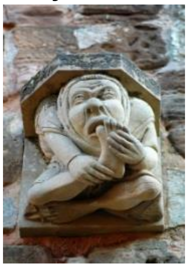

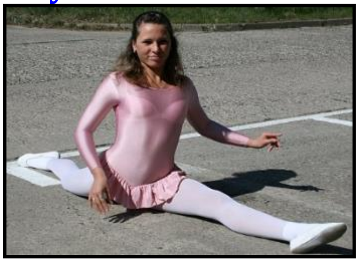

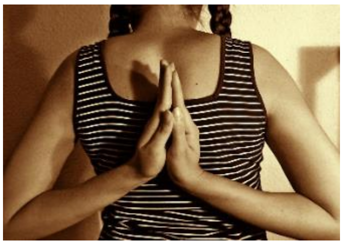

YES - NO

Continue on the Back

4. Have ever dislocated your shoulder, kneecap, or any other joint?

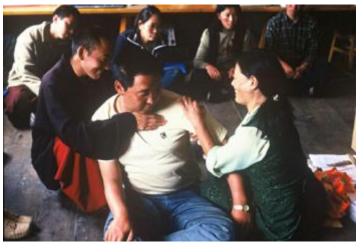

YES - NO

5. Can you easily dislocate or arch your joints? (e.g., fingers, wrist, elbows, etc.) TRY IT. Any one of them counts

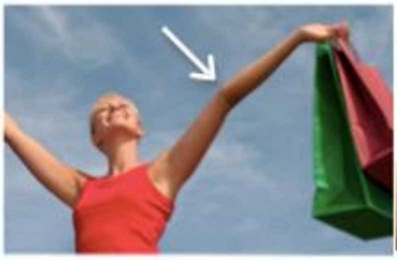

Can you bend your elbow like this? Try it

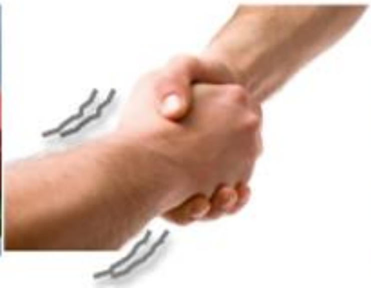

Does your wrist dislocate slightly when shaking hands?

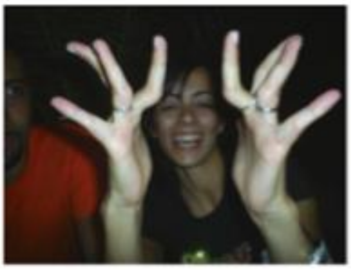

Can you bend your fingers, knees, back, etc., a lot

YES - NO

6. Do you tend to have thick or wide scars? And stretch marks on the skin? (See some examples) Just one counts as Yes.

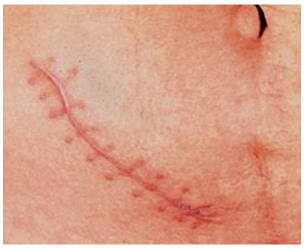

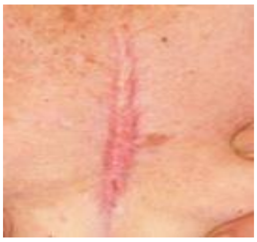

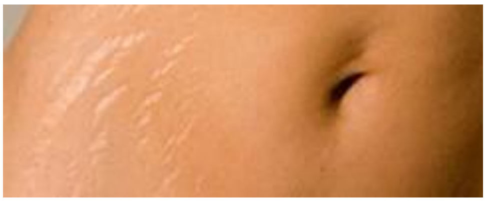

YES - NO

7. Do you get bruises, barely remembering any knocks?

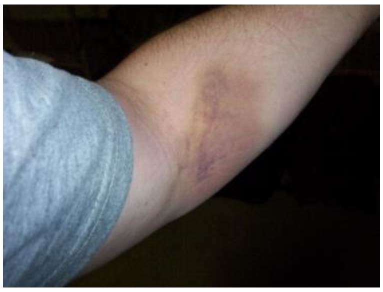

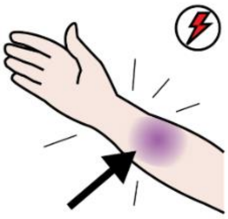

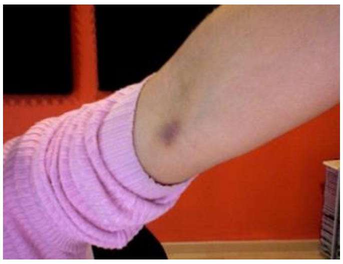

YES - NO

Thank you very much for your collaboration!

NEUROCONNECTIVE PHENOTYPE SCORE

	TOTAL
**SENSORY SENSITIVITY**	
**BODY SIGNS**	
**PAIN**	
**CHARACTERISTICS AND SENSATIONS**	
**TRENDS AND BEHAVIOURS**	
**SOMATIC DIAGNOSES**	
**PSYCHIATRIC DIAGNOSES**	
**HYPERLAXITY GUIDELINE HLX 9**	
**BEIGHTON CRITERIA**	
**Collagen Elasticity Questionnaire SHC 7**	

NEUROCONNECTIVE Model (2023)
